# Supplementary material for: The acceptability and effect of a culturally-tailored dance intervention to promote physical activity in women of South Asian origin at risk of diabetes in the Netherlands—A mixed-methods feasibility study
Source: PLoS One. 2022 Feb 25;17(2):e0264191. doi: 10.1371/journal.pone.0264191 (PMC8880860; doi:10.1371/journal.pone.0264191)
Supplement: S1 Table — (DOCX) [file pone.0264191.s001.docx]

**S1 Table – Topic-guides for interviews with Participants, Instructor, Community leader**

| **Participants** | **Instructor** | **Community leader** |
| --- | --- | --- |
| Motivation to take part, expectations | Motivation to take part, expectations | Motivation to take part, expectations |
| Experiences with the program, instructor, community leader | Experiences with the program, participants, community leader | Experiences with the program, participants, Instructor |
| Successful and less successful elements of the program | Successful and less successful elements of the program | Successful and less successful elements of the program |
| Challenges for participation, attendance, perseverance | Role of instructor (characteristics/competencies) | Role of key leader and community centre |
| Impact of program (PA behavior, PA motivation, PA self-confidence, PA goal setting, side effects) | Adherence to protocol (fidelity) |  |
| Continuation (intention, challenges, needs) | Implementation and scale up (challenges, needs) | Implementation and scale up (challenges, needs) |
|  | Transferability | Transferability |
